# A Population-Based Linked Cohort Study of Maternal Exposure to Bioclimatic Stress and Hypertensive Disorders of Pregnancy in Australia.

**DOI:** 10.23889/ijpds.v9i5.2728

**Published:** 2024-09-18

**Authors:** Amanuel Gebremedhin, Sylvester Nyadanu, Ivan Hanigan, Gavin Pereira

**Affiliations:** 1 Edith Cowan University; 2 Curtin University

## Objective

To identify critical susceptible periods for the associations between bioclimatic exposure (Universal Thermal Climate Index, UTCI) and the risks of gestational hypertension and preeclampsia.

## Approach

We linked 415,091 singleton pregnancies of 20–42 weeks’ gestation between 1st January 2000 and 31st December 2015 to UTCI exposure in Western Australia. Distributed lag nonlinear logistic regressions were used to estimate weekly-specific adjusted odds of gestational hypertension and preeclampsia for UTCI exposure from twelve weeks of preconception through pregnancy.

## Results

The analysed pregnancies included 3.7% and 2.8% cases of gestational hypertension and preeclampsia, respectively. Exposures from early pregnancy up to 30th gestational weeks were associated with greater odds of hypertensive disorders of pregnancy (HDPs) with critical susceptible periods at all exposure thresholds, particularly elevated at the 1st (10.2 °C) and 99th (26.0 °C) exposure centiles as compared to the median (14.2 °C). The most elevated weekly OR and 95% CI estimated at 99th exposure centile was 1.07 (95% CI 1.06, 1.08) in gestational weeks 8–18 for gestational hypertension and 1.10 (95% CI 1.08, 1.11) in gestational weeks 11–16 for preeclampsia. Cumulative exposures associated with HDPs with relatively higher ORs, but less precise than weekly exposures. The effects of high exposure were disproportionately elevated in both HDPs for Caucasians, non-smokers, multiparous, and mothers in moderate/low SES residences.

## Conclusions and Implications

Maternal bioclimatic exposure increases the risk of HDPs, suggesting potentially susceptible periods and populations for climate-related targeted interventions. It highlights the need for advising women on environmental risks in early pregnancy.

